# The Effects of Presenting AI Uncertainty Information on Pharmacists’ Trust in Automated Pill Recognition Technology: Exploratory Mixed Subjects Study

**DOI:** 10.2196/60273

**Published:** 2025-02-11

**Authors:** Jin Yong Kim, Vincent D Marshall, Brigid Rowell, Qiyuan Chen, Yifan Zheng, John D Lee, Raed Al Kontar, Corey Lester, Xi Jessie Yang

**Affiliations:** 1 Industrial and Operations Engineering University of Michigan Ann Arbor, MI United States; 2 College of Pharmacy University of Michigan Ann Arbor, MI United States; 3 Industrial and Systems Engineering University of Wisconsin-Madison Madison, MI United States

**Keywords:** artificial intelligence, human-computer interaction, uncertainty communication, visualization, medication errors, safety, artificial intelligence aid, pharmacists, pill verification, automation

## Abstract

**Background:**

Dispensing errors significantly contribute to adverse drug events, resulting in substantial health care costs and patient harm. Automated pill verification technologies have been developed to aid pharmacists with medication dispensing. However, pharmacists’ trust in such automated technologies remains unexplored.

**Objective:**

This study aims to investigate pharmacists’ trust in automated pill verification technology designed to support medication dispensing.

**Methods:**

Thirty licensed pharmacists in the United States performed a web-based simulated pill verification task to determine whether an image of a filled medication bottle matched a known reference image. Participants completed a block of 100 verification trials without any help, and another block of 100 trials with the help of an imperfect artificial intelligence (AI) aid recommending acceptance or rejection of a filled medication bottle. The experiment used a mixed subjects design. The between-subjects factor was the AI aid type, with or without an AI uncertainty plot. The within-subjects factor was the four potential verification outcomes: (1) the AI rejects the incorrect drug, (2) the AI rejects the correct drug, (3) the AI approves the incorrect drug, and (4) the AI approves the correct drug. Participants’ trust in the AI system was measured. Mixed model (generalized linear models) tests were conducted with 2-tailed *t* tests to compare the means between the 2 AI aid types for each verification outcome.

**Results:**

Participants had an average trust propensity score of 72 (SD 18.08) out of 100, indicating a positive attitude toward trusting automated technologies. The introduction of an uncertainty plot to the AI aid significantly enhanced pharmacists’ end trust (*t*_28_=–1.854*; P=*.04). Trust dynamics were influenced by AI aid type and verification outcome. Specifically, pharmacists using the AI aid with the uncertainty plot had a significantly larger trust increment when the AI approved the correct drug (*t*_78.98_=3.93; *P*<.001) and a significantly larger trust decrement when the AI approved the incorrect drug (*t*_2939.72_=–4.78; *P*<.001). Intriguingly, the absence of the uncertainty plot led to an increase in trust when the AI correctly rejected an incorrect drug, whereas the presence of the plot resulted in a decrease in trust under the same circumstances (*t*_509.77_=–3.96; *P*<.001). A pronounced “negativity bias” was observed, where the degree of trust reduction when the AI made an error exceeded the trust gain when the AI made a correct decision (*z*=–11.30; *P*<.001).

**Conclusions:**

To the best of our knowledge, this study is the first attempt to examine pharmacists’ trust in automated pill verification technology. Our findings reveal that pharmacists have a favorable disposition toward trusting automation. Moreover, providing uncertainty information about the AI’s recommendation significantly boosts pharmacists’ trust in AI aid, highlighting the importance of developing transparent AI systems within health care.

## Introduction

### Background

Pharmacists play a pivotal role in ensuring patients receive the correct medications as prescribed by health care providers. This involves a critical verification task, where pharmacists must match the medication dispensed in filled bottles with the prescription labels. Dispensing errors, defined as instances when patients receive the wrong drug or dosage, significantly contribute to preventable adverse drug events, leading to approximately 700,000 emergency department visits and 100,000 hospital admissions each year [1]. Several challenges contribute to these errors including, but not limited to, limitations in current technology; lack of standardized procedures; and the high cognitive workload imposed on pharmacy staff, who often manage numerous tasks simultaneously [1-4]. To enhance patient health outcomes, reduce unnecessary health care costs, and alleviate the burden on pharmacists, it is essential to develop and implement reliable tools that minimize the risk of dispensing errors [5].

Since the 1990s, the implementation of barcode scanning systems has been advocated as a means to reduce medication errors [6]. The adoption of such systems within pharmacies and broader health care environments has led to a notable reduction in medication errors [7-10]. Nevertheless, research indicates that barcode scanning systems are not immune to workarounds and human errors [11-13]. Moreover, these systems do not adequately address the challenges faced by overburdened pharmacists [14-17].

In response to these challenges, pill counting and verification or recognition systems using image classification technologies have emerged [18-21]. Innovations like Eyecon and VIVID use vision-based methods to count medications placed on the tray. More recently, advancements have been made in automated pill identification through feature engineering. For example, Yu et al [22] and Yu et al [23] proposed an automatic pill recognition method based on pill imprints, achieving an accuracy of 86.01% and 90.46%, respectively. Caban et al [18] used a modified shape distribution technique to determine the shape, color, and imprint of a pill to identify the drug. The proposed technique was evaluated with 568 of the most prescribed drugs in the United States and achieved a 91.13% accuracy.

The advent of deep learning has further enhanced the capabilities of automated pill recognition systems [5,24]. For instance, Larios Delgado et al [5] developed a pill recognition method using 2 deep learning models. They used a deep convolutional neural network model for pill blob detection to isolate the pill from the background and then passed the output to a deep learning–based classifier to identify the 5 most likely pills with 94% accuracy [5]. Similarly, Wong et al [25] proposed a deep convolutional network model and achieved a mean accuracy of 95.35%. Lester et al [24] trained a ResNet-18 deep learning model to predict the labeled features of a medication product using an image showing medication inside a filled prescription bottle. In a test set containing 65,274 images of 345 unique oral drug products, the overall macroaverage precision, that is, the mean of precision values for each class, was 98.5%.

Despite the impressive strides in model accuracy, realizing the potential of these technologies is only possible if people establish appropriate trust in them. Trust in automation, defined as “the attitude that an agent will help achieve an individual’s goals in situations characterized by uncertainty and vulnerability” [26], is one of the most crucial factors determining the use of automation [27,28]. There is a growing body of research examining people’s trust in autonomous and robotic technologies in various domains, including transportation [29-31], health care [32,33], education [34], and defense [35,36]. In addition, researchers have developed various methods to enhance people’s (proper) trust in automation or autonomy [37,38], including the use of various graphical representations [37,39-42].

For example, a military perimeter defense experiment conducted by Mercado et al [43] aimed to investigate the role of intelligent agent transparency on operator trust. Participants were tasked with selecting optimal routes for unmanned vehicles, assisted by an artificial intelligence (AI) agent. The AI agent operated at three levels of transparency: (1) basic details only; (2) basic details supplemented with reasoning and rationale; and (3) comprehensive information, including basic details, reasoning, rationale, and uncertainty indication, in a text description. They observed a positive correlation between transparency levels and participant trust. They concluded that providing operators with the agent’s reasoning process and uncertainty metrics fostered a deeper understanding of the system’s capabilities, thereby enhancing trust and increasing usability [43]. This finding emphasizes the importance of transparent AI systems in supporting effective human-machine collaboration.

Another study investigated the impact of visual explanations on human trust in machine learning systems [40]. Participants performed leaf classifying tasks with or without visual explanations. The leaf examples were presented in 2 formats: images and feature charts. Results revealed that providing visual explanations enhanced trust and confidence in participants’ decision-making. Interestingly, the feature charts were designed with intentional omissions of detailed explanations of features to prevent information overload. However, this simplification led participants to struggle to interpret the charts, and expert users expressed a need for more comprehensive feature descriptions to inform their decisions [40]. This insight reveals the importance of integrating visual explanations with a thoughtfully managed information load for appropriate human trust.

Signal detection theory (SDT) is commonly used to study trust in automation by modeling the reliability of automated systems used by human operators. SDT evaluates how AI systems distinguish signals from noise, categorizing the state of the world as either “signal present” or “signal absent.” Based on SDT categorization, AI performance results in 4 outcomes: hit (error flagged correctly), miss (error not flagged), false alarm (FA; no error, but flagged incorrectly), and correct rejection (CR; no error and no flag). Correct identifications (hit and CR) increase trust, while incorrect ones (FA and miss) decrease trust [44,45]. Research indicates that FAs typically reduce trust less than misses, prompting designers to design more liberal systems (ie, more willing to flag an error) with higher rates of FAs to ensure potential issues are flagged [46-48]. In the context of pill dispensing, FAs may lead to minor disruptions, while misses could lead to dispensing errors, indicating that a more liberal AI system prioritizing safety by minimizing misses is beneficial.

### Objectives

This study, therefore, aimed to explore pharmacists’ trust in automated pill verification technology, which is designed to assist with the critical task of medication dispensing. Specifically, we aimed to study the role of presenting AI uncertainty information on pharmacists’ trust in the system. The primary hypothesis was as follows:

- H1: Presenting AI uncertainty information of predicted probability in a visualization format will increase AI transparency, leading to enhanced pharmacists’ trust in pill verification technology.

Beyond this primary focus, we also explored how pharmacists’ trust behavior varied across different AI performance patterns categorized by SDT. Drawing from these arguments, we derived the following hypotheses:

- H2: Misses would result in a more significant decline in trust compared to FAs.
- H3: Furthermore, given the differing consequences associated with the 4 SDT patterns, we speculate that presenting AI uncertainty information might have varying effects depending on the specific type of patterns.

## Methods

### Ethical Considerations

This research was exempt from institutional review board oversight by the University of Michigan (HUM#00213493). Before participating, participants signed an electronic informed consent form, and all data were collected anonymously. Participants received US $150 upon completion of the study.

### Recruitment and Participants

**Figure 1:**
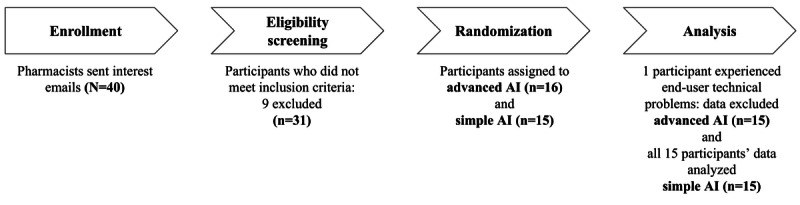
Participant recruitment timeline.

Recruitment emails were dispatched to pharmacists through the Minnesota Pharmacy Practice-Based Research Network and the University of Michigan College of Pharmacy Pharmacist Preceptor Network. To meet the inclusion criteria, pharmacists were required to (1) be licensed pharmacists in the United States, (2) be aged at least 18 years, and (3) have access to a laptop or desktop computer with a webcam. Pharmacists who (1) require assistive technology to use the computer; (2) wear eyeglasses with more than one power; (3) have uncorrected cataracts, intraocular implants, glaucoma, or permanently dilated pupils; and (4) have eye movement or alignment abnormalities (eg, lazy eye, strabismus, nystagmus) were excluded from participation in the study (Figure 1). A total number of 30 licensed pharmacists in the United States completed the study. Table 1 shows the demographic information.

**Table 1:** Participant demographic information (n=30).

Characteristic		Value	

**Age (years), mean (SD)**		39.40 (11.23)	
**Sex, n (%)**			
	Female	17 (57)	
	Male	13 (43)	
**Practice setting, n (%)**			
	Community pharmacy	15 (50)	
	Hospital pharmacy	6 (20)	
	Specialty pharmacy	1 (3)	
	Grocery store or mass merchandise pharmacy	1 (3)	
	Other	7 (23)	
**Years worked, n (%)**			
	1-5	7 (23)	
	6-10	7 (23)	
	11-20	10 (33)	
	21 or more	6 (20)	

### AI Model

The AI model used in this study is a Bayesian neural network that predicts the National Drug Code (NDC), a unique identifier assigned by the Food and Drug Administration to catalog drug products in the United States [49,50]. The Bayesian neural network used a technique known as random dropout [51], applied to a ResNet-34 convolutional neural network architecture [52] to estimate the probability associated with each NDC (ie, each class). The model produced 50 different predictions, where each prediction is a probability vector that quantifies the probabilities that an input belongs to each of the NDCs. The predicted NDC was then attained by finding the highest average probability derived from the 50 predictions.

To train the AI model, we acquired a dataset of 432,974 images from a mail-order pharmacy in the United States. This pharmacy uses a robotic system that counts pills, fills and labels the bottle, captures the images of the contents, and seals the bottle with a cap. The image dataset consists of 1 year’s worth of robot-captured images of oral medications, such as tablets and capsules, inside prescription bottles filled by the robotic system. Each image in the dataset is associated with an NDC label and various attributes of color, shape, size, manufacturer, tablet scoring, and imprint. The number of images available for each NDC varied, ranging from 3 to 12,105, with a median of 540 (IQR 257-1291). The medications featured in these datasets were classified into 12 different colors and 7 distinct shapes. The detailed classification is shown in Table 2.

**Table 2:** Percentage of medication characteristics featured in the training dataset (N=260,119).

Characteristics		Dataset, n (%)
**Colors**		
	White	109,487 (42.1)
	Yellow	32,041 (12.3)
	Pink	23,585 (9.1)
	Orange	18,541 (7.1)
	Multicolor	15,289 (5.9)
	Green	13,644 (5.2)
	Red	13,474 (5.2)
	Blue	12,452 (4.8)
	Brown	9792 (3.8)
	Purple	8184 (3.1)
	Turquoise	1858 (0.7)
	Gray	1772 (0.7)
**Shapes**		
	Round	128,947 (49.6)
	Oval	86,844 (33.4)
	Capsule	42,040 (16.2)
	Hexagon (6-sided)	1150 (0.4)
	Triangle	738 (0.3)
	Trapezoid	280 (0.1)
	Pentagon (5-sided)	120 (0)

### Experimental Testbed and Stimuli

In the experiment, participants performed a pill verification task with the help of an imperfect AI aid that recommends whether to accept or reject a filled medication. The participant’s task was to verify whether the filled medication matched the reference image. If the reference image and filled medication did not match, the correct action was to click “reject.” If the reference image and filled medication matched, the correct action was to click “accept.”

**Figure 2:**
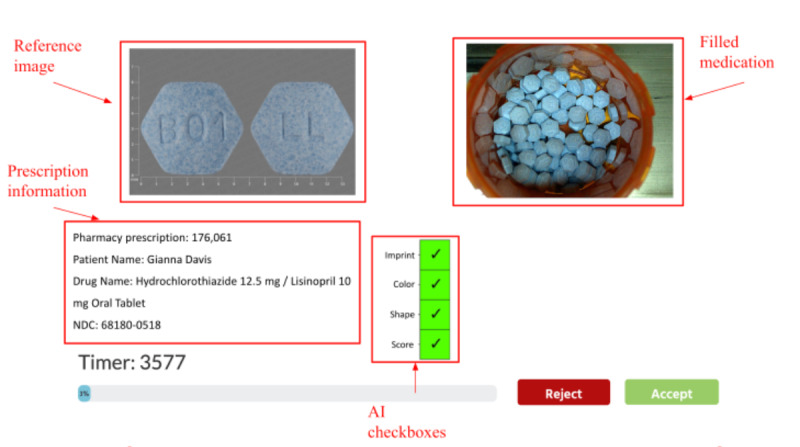
Interface for the AI aid without the uncertainty plot. Checkboxes indicate AI’s recommendation. When all 4 checkboxes are green, the AI advises to accept; otherwise, it advises to reject. AI: artificial intelligence; NDC: National Drug Code.

**Figure 3:**
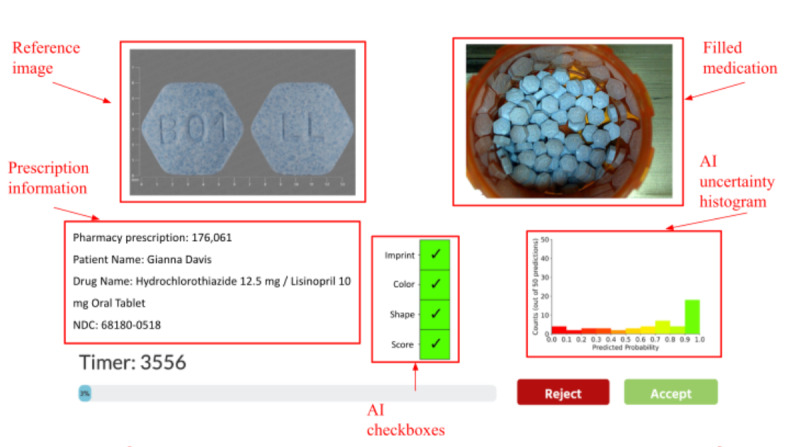
Interface for the AI aid with the uncertainty plot. In addition to Figure 2, the histogram shows the distribution of the 50 predicted probabilities associated with the predicted NDC. AI: artificial intelligence; NDC: National Drug Code.

The user interface was designed following pharmacists’ feedback from a focus group study conducted by the research team [50]. The interface displayed an image of a filled medication, a reference image, prescription information, and AI aids. There were 2 types of AI aids powered by the same AI model: one aid augmented with an uncertainty plot indicating the degree of certainty (or uncertainty) of the AI recommendation, and the other aid without this feature. Both AI aids recommend the action the pharmacist should take, using 4 checkboxes. A recommendation to accept was indicated when all four checkboxes turned green (Figure 2); otherwise, the recommendation was to reject. For the AI aid with the uncertainty plot, an additional histogram was integrated (Figure 3). The histogram displayed the distribution of the 50 probabilities for the predicted NDC, generated by the 50 predictions. The purpose of the histogram was to provide a visual representation of the certainty or uncertainty level associated with the AI’s NDC prediction.

With the help of an AI aid, participants performed a block of 100 pill verification trials. The experimental stimuli for the 100 trials, including the reference NDC and the filled medication, were carefully selected from the dataset of 432,974 images. The selection process ensured a broad representation of colors and shapes (Table 3), while blurry images were excluded to maintain clarity. To minimize learning effects, each reference NDC was intentionally shown no more than twice throughout the experiment.

**Table 3:** Percentage of medication characteristics featured in artificial intelligence–aided trials (N=100) for reference images and filled images.

Characteristics		Reference, n (%)	Filled, n (%)
**Color**			
	White	35 (35)	37 (37)
	Yellow	12 (12)	12 (12)
	Pink	8 (8)	8 (8)
	Orange	8 (8)	8 (8)
	Multicolor	3 (3)	3 (3)
	Green	6 (6)	6 (6)
	Red	3 (3)	2 (2)
	Blue	10 (10)	10 (10)
	Brown	10 (10)	10 (10)
	Purple	4 (4)	3 (3)
	Turquoise	1 (1)	1 (1)
	Gray	1 (1)	0 (0)
**Shape**			
	Round	54 (54)	51 (51)
	Oval	24 (24)	28 (28)
	Capsule	19 (19)	19 (19)
	Hexagon (6-sided)	2 (2)	1 (1)
	Triangle	0 (0)	0 (0)
	Trapezoid	1 (1)	1 (1)
	Pentagon (5-sided)	0 (0)	0 (0)

**Figure 4:**
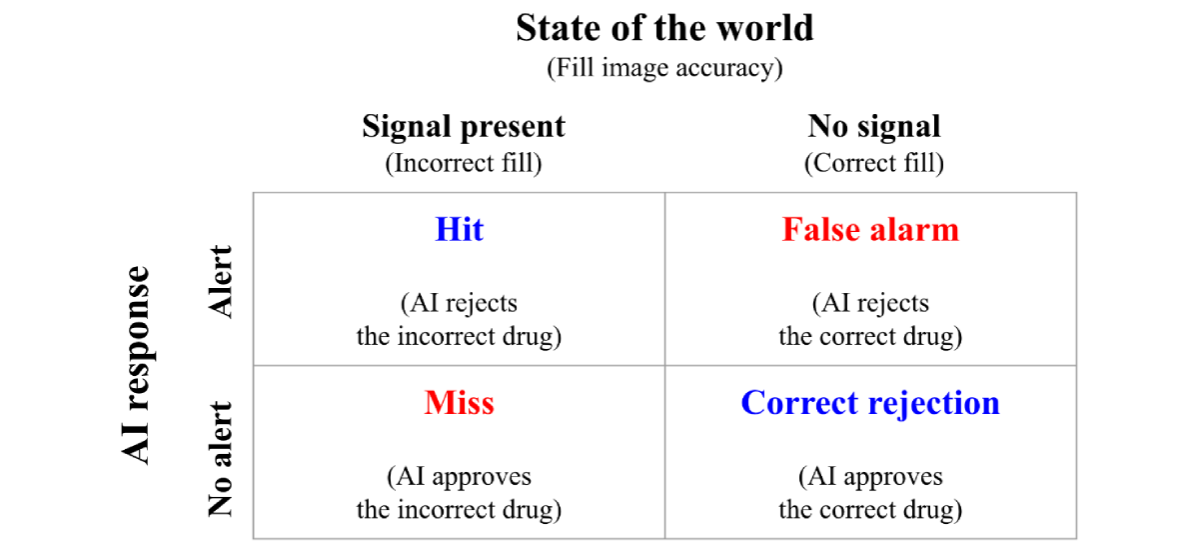
Four potential AI performance patterns according to signal detection theory. AI: artificial intelligence.

Furthermore, the AI aid was not perfect for the 100 trials, that is, it occasionally offered incorrect recommendations. Based on SDT, we mapped out the relationship between the AI’s recommendation and the true state of the world [53]. In the context of this experiment, a signal in the world was an incorrectly filled medication. The extent to which the AI recommended rejecting an incorrectly filled medication reflects its ability to detect the signal. The combination of the state of the world and the AI’s recommendation resulted in four potential outcomes: (1) the AI rejects the incorrect drug (hit), (2) the AI approves the incorrect drug (miss), (3) the AI rejects the correct drug (FAs), and (4) the AI approves the correct drug (CRs), as shown in Figure 4.

Benchmarking prior literature [54], the base rate was set to be 24%, that is, 24% of the trials contained wrongly filled medication. The AI accuracy was set as 82% to ensure that the AI would be perceived as useful while providing sufficient misses and false alarms [55]. By combining the filled image accuracy and AI accuracy, there were 60 cases of the AI approving the correct drug, 22 cases of the AI rejecting the incorrect drug, 2 cases of the AI approving the incorrect drug, and 16 cases of the AI rejecting the correct drug.

**Figure 5:**
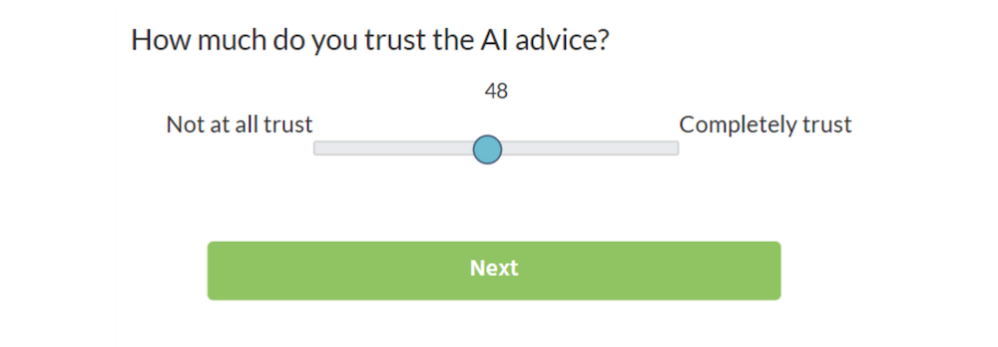
Participants reported their trust in the recognition AI on a visual analog scale. AI: artificial intelligence.

After each trial, participants received performance feedback indicating the correctness of their decision to accept or reject, as well as whether the prescription bottle was correctly or incorrectly filled (ie, “Your decision was correct. The medication was correctly filled”). Following this step, participants reported their trust in the recognition AI on a visual analog scale, with the leftmost point labeled “Not at all trust” and the rightmost point labeled “Completely trust” (Figure 5) [44,56,57].

### Experimental Design

The experiment used a mixed subjects design. The between-subjects factor was the type of AI aid, distinguished by the presence or absence of an uncertainty plot. The within-subjects factor was the four potential outcomes: (1) the AI rejects the incorrect drug, (2) the AI rejects the correct drug, (3) the AI approves the incorrect drug, and (4) the AI approves the correct drug (Figure 4).

Half of the participants used the AI aid without the uncertainty plot, and the other half used the AI aid with the uncertainty plot. Each participant completed 2 blocks of 100 trials each. One block involved using the AI aid (either with or without the uncertainty plot), and the other block required participants to perform the task manually. The order of the 2 blocks was counterbalanced. Additionally, benchmarking prior literature [45], the trial sequence was fixed for the 100 trials in each block.

As this study is focused on the pharmacists’ trust in AI, data from the manual task block were excluded from the analysis, concentrating the study’s findings on interactions involving the AI aid.

### Measures

#### Trust propensity

Before the experiment, we measured participants’ trust propensity using the 6-item survey used by Merritt et al [58]. Trust propensity is “a stable, trait-like tendency to trust or not trust others” [59], and the propensity to trust machines reflects a person’s tendency to trust machines in general rather than in a particular machine.

#### End Trust

End trust, *Trust*(*100*), is the participant’s final trust rating after interacting with the AI help scenario.

#### Average Trust

Average trust denotes the mean of moment-to-moment trust ratings collected throughout the experiment.



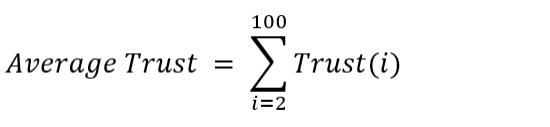



#### Trust Change

After each trial *i*, participants reported their *Trust*(*i*) in the AI. We calculated a trust change as follows.

Trust change (*i*) = Trust(*i*) – Trust(*i* – 1), where *i*=2, 3, ..., 100.

Since the moment-to-moment trust was reported after each trial, 99 trust changes were obtained from each participant.

### Experimental Procedure

The experiment was conducted remotely with interested participants who met the inclusion criteria. Each interested individual was phone screened to determine their eligibility. Before each experiment, participants had a brief web-based meeting with a member of the study team to ensure that the physical environment, including lighting conditions, was suitable for the experiment. Subsequently, the pharmacists were directed to Labvanced’s website on their computer and presented with a 15-minute video tutorial that explained how to perform a simulated medication verification task using the testbed interface. Pharmacists were informed that the objective of the task was to determine whether an image of a filled medication bottle matched a known reference image. The tutorial also explained the 2 AI aids.

Before engaging in the verification task, participants were directed to complete a demographics survey and a trust propensity survey [58]. Additionally, they went through a set of calibration procedures for the eye-tracking software. Participants then completed a block of 100 verification trials using an AI aid—either with or without the uncertainty plot—and another block of 100 verification trials manually, conducted in a counterbalanced order. Upon completion of the 200 trials, participants filled out a postexperimental survey and answered nonmandatory free-response feedback questions. All participants finished each block within the time limit of 60 minutes.

After completing all the tasks and surveys described earlier, participants were invited to a 30-minute debriefing session with one of the study team members. Study team members described 6 concepts of automation evaluation: observability, predictability, directing attention, exploring solution space, adaptability, and calibrated trust [60], and provided an example scenario for each concept. After each description and example, the participants were asked to provide their thoughts on how the concept relates to our system.

### Statistical Analysis

Participants’ trust propensity, end trust, and trust change when using both types of AI aids were analyzed. First, we conducted a descriptive analysis of the participants’ trust propensity. Then, to test our directional hypothesis that AI aids with uncertainty will result in higher end and average trust, we conducted a 1-tailed *t* test. Finally, we analyzed how trust increased and decreased after participants experienced each of the 4 AI performance patterns using mixed-linear models with random intercept. Regression 2-tailed *t* tests were conducted to compare the means between the 2 AI aids for each AI performance pattern. The Kenward-Roger method was used to estimate degrees of freedom. Mixed model (generalized linear models) tests were conducted using the R (version 4.2.2; R Foundation for Statistical Computing) *lme4* package [61]. All statistical significance was determined at the ɑ=.05 level and analyses were carried out using R statistical software [62].

## Results

### Trust Propensity

Participants had an average trust propensity score of 72 (SD 18.08) out of 100, indicating the participants generally had a positive attitude toward trusting automated technologies. There was no significant difference between the 2 AI aids (t_28_=–0.854; *P*=.20; Cohen *d=*.312).

### End Trust Toward AI Aid

**Figure 6:**
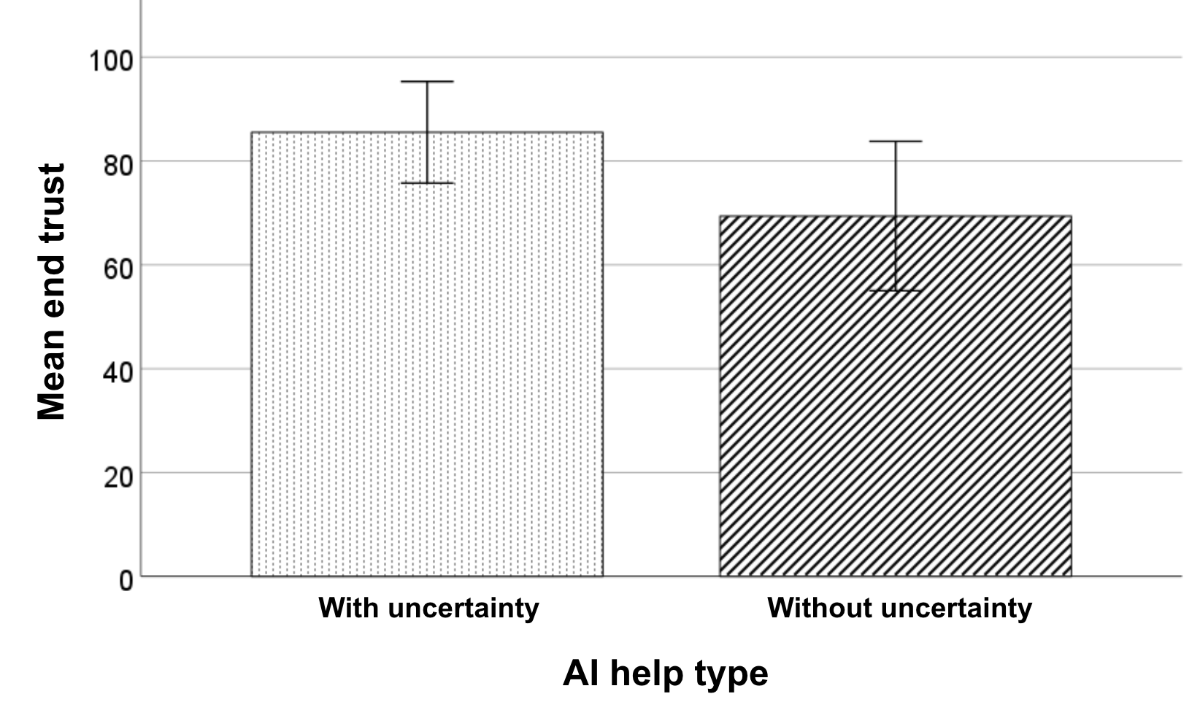
Mean end trust by AI aid help type (with or without the uncertainty plot). The error bars represent a 95% CI. AI: artificial intelligence.

The 1-tailed *t* test indicated that participants trusted the AI aid with the uncertainty plot significantly more than the AI aid without the plot at the end of the experiment (t_28_=–1.854; *P*=.04; Cohen *d*=–.677; Figure 6).

### Average Trust Toward AI Aid

**Figure 7:**
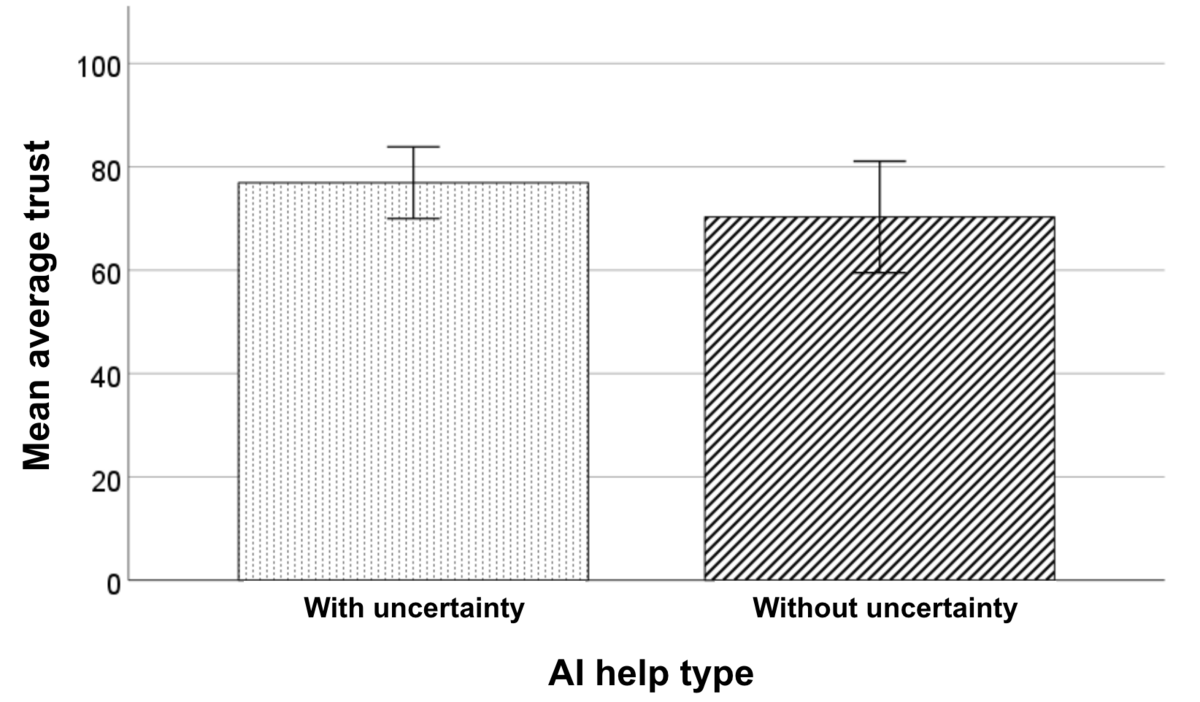
Mean average trust by AI aid help type (with or without the uncertainty plot). The error bars represent a 95% CI. AI: artificial intelligence.

Participants showed a slightly higher average trust in the AI aid in the with-uncertainty condition (mean 76.92, SD 13.42) than in the without-uncertainty condition (mean 70.29, SD 20.88; Figure 7). However, the difference did not reach statistical significance (t_28_=–1.036; *P*=.16; Cohen *d*=–.378).

### Trust Change

**Figure 8:**
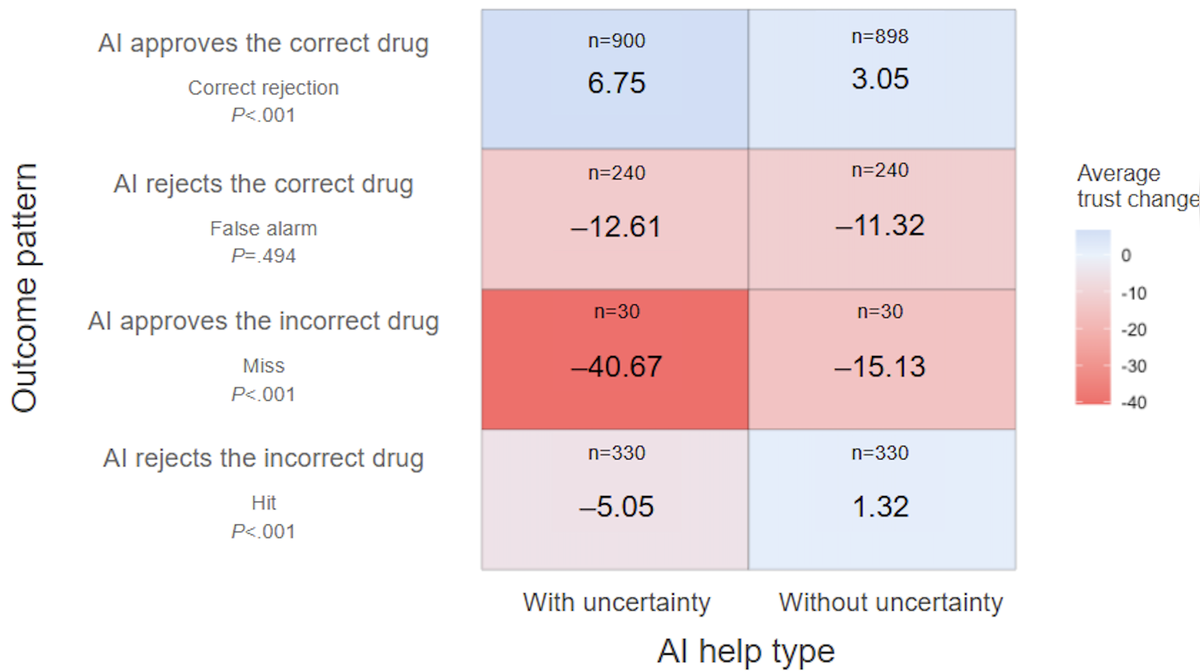
Trust change for different AI performance patterns. Red shades represent negative trust change, and blue shades represent positive trust change.

Figure 8 shows the trust change after participants experienced the 4 AI performance patterns. When the AI approved the correct drug, there was a significantly greater trust increment when participants used the AI aid with the uncertainty plot compared to the one without (t_78.98_=3.93; *P*<.001; Cohen *d*=.214); When the AI approved the incorrect drug, there was a significantly greater trust decrement using the AI aid with the uncertainty plot (t_2939.72_=–4.78; *P*<.001; Cohen *d*=.712). Interestingly, when the AI rejected the incorrect drug, we observed a decrement of trust for participants using the AI aid with the uncertainty plot (t_509.77_=–3.96; *P*<.001; Cohen *d*=.312). When the AI rejected the correct drug, both AI help types showed a decrease in trust and there was no statistical difference between them (t_856.57_=–0.68; *P*=.49; Cohen *d*=.045). Overall, participants using the AI aid with the uncertainty plot displayed a large magnitude of trust adjustment. In addition, we observed a significant “negativity bias” in that the magnitude of trust change when the AI made an error (ie, the AI approves the incorrect drug or the AI rejects the correct drug) was significantly larger than the magnitude of trust adjustment when the AI provided correct recommendations (generalized linear model test; *z*=–11.30; *P*<.001).

To examine variation in pharmacists’ trust behavior across different AI performance patterns categorized by SDT, we initially combined the data from both the with and without uncertainty help scenarios. Trust decreased significantly more when the AI approved the incorrect drug compared to when the AI rejected the correct drug (t_509_=–4.687; *P*<.001; Cohen *d*=.475). This trend was observed in the with-uncertainty AI help scenario (t_254_=–4.91; *P*<.001; Cohen *d*=.758). However, in the without-uncertainty AI help scenario, although there was a greater trust decrease in trust when the AI approved the incorrect drug than when the AI rejected the correct drug, the difference was not statistically significant (t_254_=–1.14*; P*=.255; Cohen *d*=.014).

**Figure 9:**
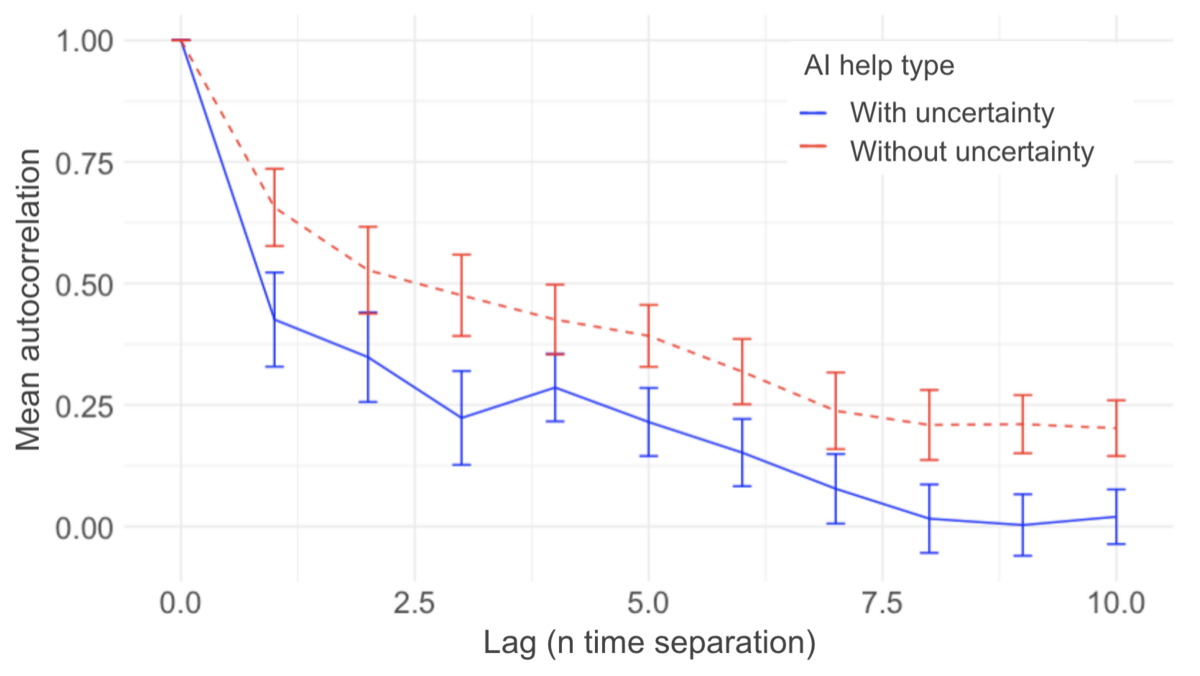
Autocorrelation of trust as a function of time separation. The blue solid line represents AI aid with the uncertainty plot, and the red dashed line represents the AI aid without the uncertainty plot. The error bars represent 2 SEs. AI: artificial intelligence.

As we measured participants’ trust toward AI continuously, we calculated the autocorrelation between the trust ratings. Autocorrelation measures the relationship between a variable’s current value and its past values in a time series. Figure 9 shows the mean autocorrelation as a function of time separation between the ratings. For both AI aids, the correlation decreased as the time separation increased. The AI aid with the uncertainty plot had a lower mean autocorrelation compared to the aid without the uncertainty plot (Figure 9).

## Discussion

### Principal Findings

This study aimed to investigate pharmacists’ trust in automated pill verification technology and how the presentation of AI uncertainty information would influence them. Overall, the findings revealed that pharmacists have a favorable disposition toward trusting automation, and including the AI’s uncertainty information increases pharmacists’ trust in the AI recommendation *(*Figure 6*)*.

### Comparison With Prior Work

The propensity to trust automation refers to an individual’s general inclination to trust automated or autonomous systems, shaped by their past experiences and future expectations [58,59]. Research has shown that levels of trust propensity vary among individuals. For example, an early study by Merritt et al [58], which included 69 college students (average age of 25 years), found an average trust propensity of 3.56 (SD 0.6) on a 7-point Likert scale [59]. More recently, Montag et al [63] surveyed 289 participants aged between 18 and 70 years and reported their propensity to trust automation to be 4.98 (SD 1.06) after converting to a 7-point Likert scale. Similarly, Miller et al [64] reported a trust propensity score of 4.97 (SD 1.21) from a smaller cohort of 28 participants aged between 18 to 60 years. Another investigation by Yang et al [65] with 75 adults (mean age 23.0) split into 3 groups reported trust propensity scores of 72.6 (SD 14.8), 69.4 (SD 10.4), and 69.4 (SD 14.4), equivalent to average scores of 5.08 (SD 1.89), 4.86 (SD 1.62) and 4.86 (SD 1.86) on a 7-point Likert scale.

In line with these findings [63-66], our study revealed that pharmacists generally have a favorable disposition toward trusting automation, with an average rating of 72 (SD 18.08) on a 100-point scale, or 5.03 (SD 2.09) on a 7-point Likert scale. This positive attitude may be attributed to the frequent use of automated technologies, such as barcode scanners and pill counters in their daily work [6,7]. Additionally, as expected, no significant difference was observed between the groups using different AI aids because the participants were randomly assigned to use either of the AI aids.

Examining pharmacists’ end trust, our findings reveal that the AI aid with the uncertainty plot significantly enhanced the end trust scores. We attribute this enhancement to the increased transparency achieved through the presentation of a histogram showing the distribution of 50 predicted probabilities. While AI advancements promise to improve human performance, a prevailing issue is the perception of AI as a “black box.” This lack of transparency contributes to a lack of trust in AI and can undermine team performance [66-68]. The higher end trust observed in participants using the AI aid with the uncertainty plot indicates that making the AI more transparent by revealing its decision-making process can foster a higher level of trust in automation. Participants 14 and 24 captured this sentiment well stating: “As soon as the uncertainty plot became red and yellow, I slowed down, which could be helpful because sometimes slowing down when there is uncertainty, just knowing there’s uncertainty, is enough” (P14) and “if the uncertainty plot was all green bar and AI thought it was doing a 100% accurate job then it was easier to make my decision” (P24).

**Figure 10:**
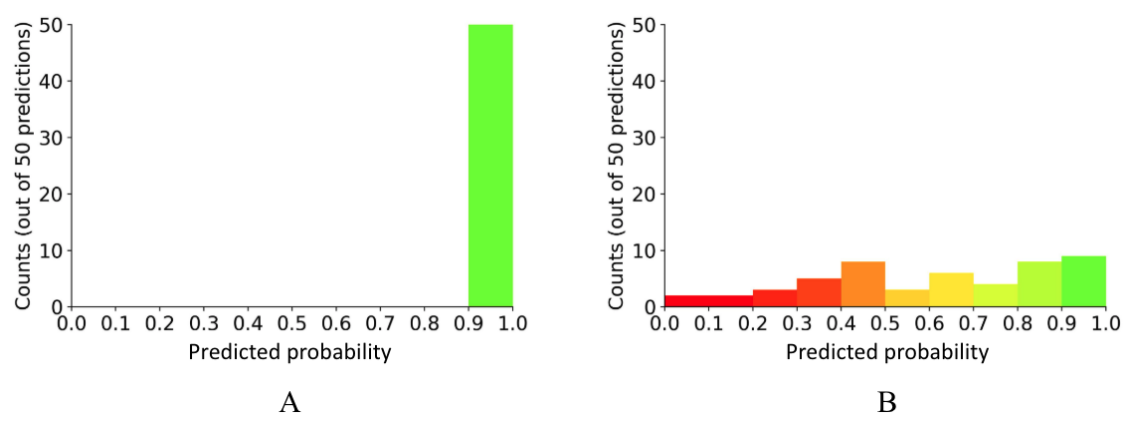
Uncertainty plots with (A) narrower and (B) wider IQRs. IQR is a measure of statistical dispersion, or how spread out the data points are.

Regarding the dynamics of trust, that is, moment-to-moment trust change, when AI approved the correctly filled bottle, we noted trust increments for both AI aids. Furthermore, the inclusion of uncertainty information led to a larger increment in trust compared to when such information was absent. When the AI mistakenly approved the incorrect drug, we observed a significant trust decrement for both AI aids, potentially attributed to the adverse outcome associated with the wrong medication. Furthermore, the trust decrement was significantly larger when the uncertainty information was shown. This pronounced trust decrement could have resulted directly from the distribution of the histogram: participants were shown a histogram indicating a high level of certainty (Figure 10A). Therefore, participants may have perceived the error made by the AI aid as a “confident” error and therefore reduced their trust even more. Studies examining likelihood alarms reported that highly likely alarms (ie, “confident” alarms) engender a greater decline in momentary trust upon automation failures [57,69].

Our study also offers additional validation of prior findings regarding the SDT modeling of trust [47,48]. When the AI mistakenly approved the incorrect drug (miss), a greater trust decrease appeared compared to when the AI rejected the correctly filled bottle (FA). However, the difference was statistically significant only in the with-uncertainty AI help type. This finding may provide further evidence that “confident” AI errors lead to a greater trust decrement [57,69].

**Figure 11:**
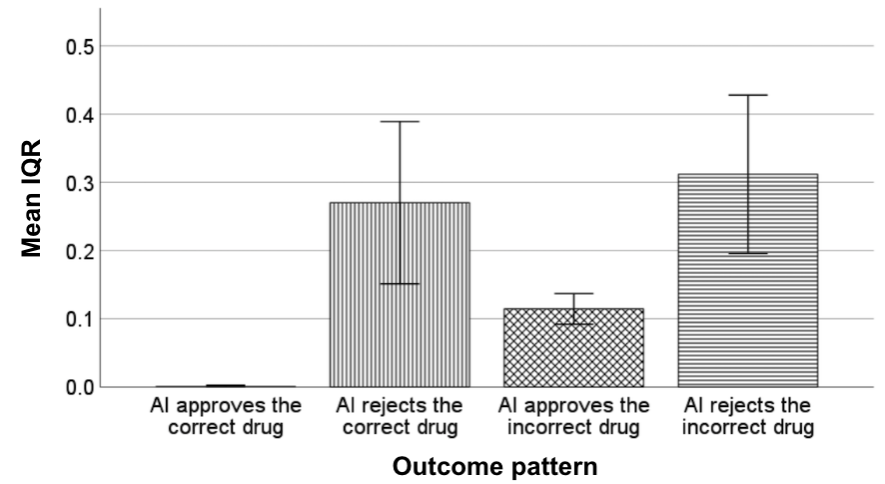
Mean IQR by outcome pattern. The error bars represent a 95% CI. AI: artificial intelligence.

Intriguingly, when the AI rejected an incorrectly filled bottle, the absence of uncertainty information resulted in an increase in trust, whereas the presence of such information led to a decrease in trust. Such contrasting results could have stemmed from the uncertainty plots influencing the participants’ decision-making process. When the AI approved the correct drug, all uncertainty plots presented to participants showed a consistent solid green bar (Figure 10A). However, when the AI rejected the incorrect drug, the IQR of the uncertainty plot was broader, indicating the lack of certainty (Figure 11). A total of 16 (73%) out of 22 uncertainty plots displayed a wider spread with mixed color bars (Figure 10B). This ambiguity might unintentionally cause the human participants to doubt the capability of the AI aid with the uncertainty plot, resulting in a decrease in their trust, as evidenced by P18’s statement: “When the checkboxes were not all green and the histogram had a bunch of colors (variability), I was even less trusting of the AI tool.” This perspective is also supported by P25, who noted: “When the uncertainty plot had some red, it made me double check and decreased my confidence for sure.”

Finally, when AI rejected the correctly filled bottle, trust decrements occurred, with no significant differences between the 2 AI aids. If this circumstance happened in the real world, the pharmacists would reinspect the filled prescription. It will likely lead to an increased workload, fatigue, and stress, which could potentially lead to a lower quality of work and a higher frequency of errors [70,71]. However, P21 offered a contrasting viewpoint that more flagging would be better than AI approving the incorrect, highlighting: “I didn’t lose as much trust when AI rejected the correct drug. I feel like AI should be there as a cautionary tool.”

The observed trends in the moment-to-moment dynamics of trust indicate a greater degree of trust adjustment when participants were assisted by the AI with the uncertainty plot. This observation is further confirmed by the autocorrelation analysis. Specifically, the trust autocorrelation plot (Figure 9) reveals a lower autocorrelation between trust ratings when the uncertainty information was presented. This suggests that current trust levels were less influenced by past trust levels, implying more significant changes in trust from moment to moment. Pharmacists relied less on previous trials and more on the information presented in the present trial, highlighting the advantages of a more transparent display.

In addition, for both AI aids, participants displayed a larger trust decrement due to incorrect automation predictions. Even though these observations may seem alarming initially, they align with the prior literature addressing negativity bias. The study suggests that failure in automation typically has a more significant negative impact on trust than a positive impact from successful automation [56,65].

### Limitations and Future Directions

We acknowledge several limitations of this study and propose directions for future research. First, as a pioneering investigation in this domain, we did not strictly control the interquartile range of the uncertainty plot (Figure 11). Future investigation should systematically examine the effects of presenting different distributions within the uncertainty plot on pharmacists’ trust. Exploring similar variations in the IQR among different outcome patterns could provide a deeper understanding of how outcome patterns could impact trust while avoiding potential confounding factors.

Second, the uncertainty plot used in this study displayed the distribution of only 1 (2%) out of 50 probabilities for the predicted NDC. Future research should consider incorporating additional contextual information to enhance the interpretability of these distributions. A notable challenge identified was that users unfamiliar with statistical representation found the uncertainty plot difficult to understand. P11 suggested simplifying the uncertainty presentation because “it would be a little bit too much for some people not as comfortable with statistics or technology.” Nonetheless, there is potential for pharmacists to become more comfortable with these plots with prolonged use and training, as evidenced by P30’s remark: “I started skeptical because it’s something I’m not familiar with, but as I got more examples of it, my trust built up quickly.” Consequently, researchers should continue to develop alternative visualization techniques that provide a more comprehensive and intuitive representation of the AI’s uncertainty, while maintaining a low complexity to accommodate users with varying degrees of statistical proficiency.

Third, this exploratory study was limited by a small sample size, which may have impacted the statistical power of the analyses on trust propensity, end trust, and average trust. This limitation likely contributed to the lack of significance observed in some results. However, despite being underpowered, our analysis still revealed a significant difference in end trust, indicating a large effect size. Regarding trust change, to maintain the AI’s perceived usefulness while still providing enough examples of both misses and FAs, we incorporated only 2 trials of the AI approving the incorrect drug (miss) and 16 trials of the AI rejecting the correct drug (FA), setting the accuracy at 82%. The statistical power to detect significant differences may have been compromised by the small sample sizes. Future research should aim to include larger participant pools to enhance the generalizability and robustness of the findings.

Finally, this study only focused on pharmacists’ trust and trust change and did not include the analysis of accuracy and reaction time. Even though focusing on trust alone is an accepted practice [53], a more comprehensive analysis linking performance with trust would likely reveal the relationship between performance and trust calibration.

### Conclusions

Dispensing errors are significant contributors to adverse drug events, which lead to considerable health care expenses and harm to patients. Despite progress made in developing automated technologies to aid pill verification, pharmacists’ trust in these systems has not been thoroughly investigated. Our research represents an initial exploration into pharmacists’ trust in automated pill verification technology, marking a significant step in understanding the integration of such systems into health care settings.

Our findings reveal that pharmacists have a favorable disposition toward trusting automation, which can likely be attributed to their frequent use of automated technologies in their daily work. Moreover, providing uncertainty information about the AI’s recommendation significantly boosts pharmacists’ trust in the AI aid, highlighting the importance of transparency in AI development. The dynamics of trust vary depending on the AI’s performance. Pharmacists using the AI aid with the uncertainty plot had a significantly larger trust increment when the AI approved the correct drug and a significantly larger trust decrement when the AI approved the incorrect drug*.* Intriguingly, the absence of the uncertainty plot led to an increase in trust when the AI correctly rejected an incorrect drug, whereas the presence of the plot resulted in a decrease in trust under the same circumstances. In addition, a pronounced “negativity bias” was observed, where the degree of trust reduction when the AI made an error exceeded the trust gain when the AI made a correct decision.

## Abbreviations

AI: artificial intelligence
CR: correct rejection
FA: false alarm
NDC: National Drug Code
SDT: signal detection theory

## Data Availability

The deidentified data produced in this study are available upon reasonable request through a data use agreement with the University of Michigan.
